# Integrating Kalman filter noise residue into U-Net for robust image denoising: the KU-Net model

**DOI:** 10.1038/s41598-024-74777-8

**Published:** 2024-10-09

**Authors:** S. Soniya, K. C. Sriharipriya

**Affiliations:** School of Electronics Engineering, Vellore Institute of Technology, Vellore, Tamilnadu 632014 India

**Keywords:** Image denoising, U-Net, Kalman filter, Gradient estimation, KU-Net, Computational models, Data processing, Image processing, Machine learning

## Abstract

In low-level image processing, where the main goal is to reconstruct a clean image from a noise-corrupted version, image denoising continues to be a critical challenge. Although recent developments have led to the introduction of complex architectures to improve denoising performance, these models frequently have more parameters and higher computational demands. Here, we propose a new, simplified architecture called KU-Net, which is intended to achieve better denoising performance while requiring less complexity. KU-Net is an extension of the basic U-Net architecture that incorporates gradient information and noise residue from a Kalman filter. The network’s ability to learn is improved by this deliberate incorporation, which also helps it better preserve minute details in the denoised images. Without using Image augmentation, the proposed model is trained on a limited dataset to show its resilience in restricted training settings. Three essential inputs are processed by the architecture: gradient estimations, the predicted noisy image, and the original noisy grey image. These inputs work together to steer the U-Net’s encoding and decoding stages to generate high-quality denoised outputs. According to our experimental results, KU-Net performs better than traditional models, as demonstrated by its superiority on common metrics like the Structural Similarity Index (SSIM) and Peak Signal-to-Noise Ratio (PSNR). KU-Net notably attains a PSNR of 26.60 dB at a noise level of 50, highlighting its efficacy and potential for more widespread use in image denoising.

## Introduction

Image noise reduction has already been accomplished with neural networks. The most widely used networks belong to a particular class called convolutional neural networks (CNNs), which are useful for a kind of tasks like handwritten digit recognition and traffic sign recognition^1^. Deep Learning in several image processing domains, and convolutional neural networks are rapidly gaining traction. In the field of image processing, image denoising is a crucial procedure. Eliminating noise from a corrupt image while preserving its essential elements is known as image denoising. Degradation function and additive noise have an impact on the observed image in most image processing issues. $\:{Z}^{{\prime\:}}\left(x,y\right)=Z\:\left(x,y\right)+a\:\left(x,x\right)$ Where $\:Z{\prime\:}(x,y)$ is the observed image, $\:Z\:\left(x,y\right)$ is a clean image and $\:a\:\left(x,x\right)$ is additive noise^2^. The development of effective solutions to address training challenges and improve performance has been the focus of extensive research recently. The simpler denoising problem has received the most attention, leading to algorithms that are frequently not easily expanded to handle the more challenging deblurring problems.

With the advent of neural networks came the proposal of numerous approaches based on experience and machine learning, which frequently call for manually adjusting parameters and solving challenging optimization problems. Convolutional neural networks (CNN) have also been thoroughly researched for denoising an image in the interim, and the first state-of-the-art model of CNN, DnCNN was created. Many denoising models in the CNN development process, like MWCNN^3,4^ and DHDN^5^, have incorporated the U-Net architecture^6^ for its consideration of multiscale features. However, models that perform better typically require more parameters, and their PSNR only increases slightly. Unfortunately, the prior related models’ attention was frequently drawn to the internal architecture of each U-Net rather than the connections between U-Nets, severely limiting their effectiveness. Dense Dense U-Net (DDUNet), a cascading U-Nets structure with multi-scale dense processing, was proposed as a way to further strengthen the connections between U-Nets^7^. Further, the U-net model has been designed with additional features, they are U-net with group normalization, Dense U-Net, and residual U-Net concatenated with input and output of encoder and decoder. Utilizing the feature of U-Net provides better performance and robustness in the denoising process but it requires more parameters to upgrade the network^8^.

Improvements have been made to the U-Net network’s skip-connection technique to ensure that it performs better when denoising. Additionally, the network can achieve deeper performance improvement when residual mapping is used in U-Net networks^9^. To enhance the visibility of fuzzy images, use a U-Net-based residual network (URNet). By connecting dilated convolution with standard convolution, the encoder module of URNet can extract more detailed image features by expanding its receptive field through hybrid convolution. At the intersection of the encoder and decoder modules, the URNet incorporates multiple ResNet building blocks. This stops the vanishing gradient from causing a decline in network speed^10^. Additionally, current denoising techniques either require extra information on noise types and levels or assume that the image’s noise is a specific type, such as Gaussian noise. Denoising in practical applications is limited by a dedicated deep learning model’s ability to only denoise a single, specific type of noise using the noise details provided at the input stage.

Despite their effectiveness, many state-of-the-art image-denoising models have deep architectures and many parameters, making them extremely complex. The accessibility and practicality of these models are limited, especially in environments with limited resources, due to their significant computational requirements. To close this gap, KU-Net provides a more effective, less complex architecture that achieves competitive performance without requiring an excessive number of resources. This increases the availability and deployability of advanced image denoising for real-time applications and devices with low processing power. Current models tend to concentrate only on direct mappings of input to output, excluding the use of external noise estimation methods that could improve the network’s learning ability. KU-Net presents a novel method to improve the learning capacity of the network by incorporating noise residue from a Kalman filter. By adding another layer of noise estimation, the Kalman filter helps the network better distinguish between noise and true image content.

The main contributions of our work are mentioned as follows:

- This work is noteworthy for introducing Kalman filtering into the U-Net architecture. The network employs the Kalman filter to estimate noise residues, which it then uses to achieve more accurate denoising.
- Gradient information is explicitly incorporated into the KU-Net model to enhance edge and texture preservation during the denoising process.

KU-Net has proven to perform better in image-denoising tasks through extensive experiments, especially when the noise level is higher. Even with a small training dataset, the model delivers better PSNR and SSIM values than several State-of-the-art techniques. KU-Net demonstrates that complex models are not always necessary to achieve high-quality denoising, as evidenced by its competitive or even superior performance despite its simpler architecture.

## Related study

Given the abundance of noise in imaging systems, a great deal of research has been done on their statistical properties. Comprehensive analyses have been supplied. This related survey elaborated on different methods from traditional to recently developed models. Firstly, look into the BM3D method, a very traditional and complex method that achieves a higher performance, Three-dimensional filtering and block matching were applied to improve sparsity for image denoising^11^. The author proposed TNRD, which is an adaptable learning framework to quickly and efficiently create methods for a range of image restoration issues. Learning optimal nonlinear reaction-diffusion models is the foundation of their methodology. Although TNRD has produced encouraging results in terms of closing the gap between computational efficiency and denoising quality, their effectiveness is intrinsically limited to the predetermined forms of previous^12^. Images distorted by spatially variant AWGN exhibit favorable performance when processed by FFDNet. A wide uniform input noise level of FFDNet can smooth out the information in the low noise level region while also effectively removing strong noise. Conversely, the denoising outcome using an appropriate non-uniform noise level map eliminates strong noise while preserving image information^13^. With the hidden layer operations, DnCNN implicitly eliminates the latent clean Image. The batch normalization methodology is further presented to improve and stabilize DnCNN’s training performance. Results show that residual learning and batch normalization can help each other out and that integrating them can improve denoising performance and speed up training^14^. When it comes to eliminating strong noise, FFDNet outperforms DnCNN due to its larger receptive field, while DnCNN’s superior modeling capacity makes it better for denoising images with lower noise levels.

The author focussed on a long-standing issue in many low-level vision applications is image restoration (IR), given its great practical value. To solve other inverse problems, this paper trains a set of efficient and quick CNN denoisers and incorporates them into a model-based optimization technique called IRCNN. CNN and Half quadratic splitting (HQS) is used in (IRCNN) to combine model-based and learning-based image denoising techniques^15^. IRCNN and DnCNN models can yield good results, but their capabilities are restricted. They perform poorly in situations where the accuracy of the noise estimation is low, and they can only be tailored for a particular type of noise.

Also, to improve the ability of the learned feature to represent noise and reduce it, DudeNet leverages two networks to extract a variety of features. DudeNet’s sparse mechanism, which extracts both global and local features and fuses them to obtain salient features to restore clear details for complex noisy images, can help trade denoising performance for processing speed in an efficient manner^16^. In some of the articles concentrated on preserving the edge and detail information, multi-feature extracting CNN with concatenation (McCNN) can improve the viewing quality of the denoised image. After extracting several characteristics from the input image and cascading them into a structure of the forward network, the McCNN exploits different-sized convolutional kernels. Five nonlinear mapping modules make up the forward network structure, and it is through these modules that more complex features and textures are extracted^17^.

Throughout the past few decades, several models have been used for picture-prior modeling to achieve the goal of image denoising. Here author uses an attention mechanism for image restoration, (ADNet) that can extract noise details from the complex background. Additionally, they increased the receptive field size using dilated convolutions, which allowed them to obtain more global context details without using more computational power^18^. Additionally, researchers have used asynchronous and greedy algorithms to balance performance and efficiency. It has also been demonstrated that improving activation functions and creating new network topologies produce superior denoising results. In^19^ Author created a BRDNet architecture to address an internal covariant shift problem and a small mini-batch problem by combining two networks to raise the width of the network. In^20^ a network MLFAN as a multilevel feature attention network gives prior knowledge about the texture information of an image and also provides better performance in the denoising process. The Fully Convolution Network (FCN) serves as the foundation for U-net’s development. Similar architectures are constructed for segmentation purposes; to enhance the segmentation performance, the model connects the intermediate decoder (down-sampling path) and the coder (up-sampling path) to integrate fine detail in the DRU-Net model. DRU-net: a biomedical image segmentation technique based on a U-net variant with a deformable encoder and reshaping up-sampling convolution decoder^21^. In^22^ presented a deep convolutional coding-decoding denoising network. This network’s convolutional layer and deconvolutional layer are connected via a skip connection structure. The gradient explosion issue and the partial lack of detailed information during feature propagation are partially resolved by this structure in addition to increasing gradient propagation efficiency from top to bottom. To optimize contrast enhancement parameters, this paper presents a gray-scale contrast enhancement algorithm that combines the Artificial Electric Field Algorithm (AEFA) with the Incomplete Beta Function (IBF). It can be difficult and time-consuming to precisely adjust the controlling parameters of IBF, but doing so is essential to its effectiveness^23^.

Numerous domains have advanced in recent deep learning models. Image stenography using transforms was specifically covered in^24–28^, followed by^29] and [30^ on breast cancer detection. Real-time applications can benefit from those methods.

With a range of noise models and different techniques, many methods have been developed in the last few years for denoising natural images. For mixed noise removal in natural digital images^31^, uses a combination of traditional diffusion-based methods and neural networks. When combined with pulse-coupled neural networks, the well-known Perona-Malik smoothing technique improves mixed noise reduction. Using GANs and semi-soft thresholding^32^, presents a method for Gaussian noise reduction. This application of GANs, which have shown great success in producing clean images, provides a sophisticated framework to deal with the randomness of Gaussian noise, and the semi-soft thresholding technique improves noise suppression without going overboard with smoothing.

Image denoising using a wavelet-based method in conjunction with dictionary learning is covered in^33^. This method is appropriate for structured and repetitive noise patterns because it groups similar patches of the image and applies sparse representation techniques, which results in effective noise reduction while preserving important image details, particularly in high-frequency components.

**Table 1:** Addressing pros and cons of few state-of-the-art methods.

Method	BM3D ^11^ 2007	TNRD ^12^ 2016	FFDNet ^13^ 2018	DnCNN ^14^ 2017	Dual CNN ^16^ 2021	Attention-Guided CNN ^18^ 2020	MLFAN ^20^ 2023
Pros	Extremely efficient at different decibel levels, traditional, and extensively used	Framework that is trainable and adaptable to various restoration tasks	Quick, adaptable, and able to tolerate a range of noise levels without requiring retraining	Deep learning and residual learning CNN execute well	Feature extraction is improved by dual-branch design	Enhances detail preservation with the attention mechanism	Strengthening multi-level feature attention with texture prior
Cons	Costly to compute and less successful when dealing with structured noise	Requires precise parameter adjustment; noise type may have an impact on performance	GPU may be needed for real-time applications, as it is still quite complex	Large datasets are needed for training, and computation is expensive	More parameters to train and a more complex model	Higher processing overhead as a result of the attention layers	Comparatively new, and needs more testing on a variety of datasets

Table 1 summarizes, the advantages and disadvantages of the few State-of-the-art methods. As of right now, deep learning-based techniques like DnCNN, FFDNet, and MLFAN are among the state-of-the-art image-denoising methods that frequently require extremely intricate architectures. The computational load of these approaches is greatly increased even though they are effective because they usually call for a large number of parameters, deep layers, and complex elements like attention mechanisms. These models’ great complexity makes them challenging to use in real-time or resource-constrained situations, in addition to requiring significant computational resources (often GPUs).

By contrast, our KU-Net proposal seeks to provide novel features like gradient information and the Kalman filter with a more straightforward architecture. The emphasis on preserving simplicity implies that it may occupy a region between high performance and moderate complexity, possibly providing superior computational efficiency over the more complicated approaches, such as MLFAN, while still producing competitive results.

## Methods and materials

In the field of image processing, the U-Net architecture has grown to be indispensable, especially for tasks like segmentation, denoising, and restoration. U-Net was initially developed for biomedical image segmentation and was intended to function well with a small amount of training data. The main objective was to design an architecture that could accurately analyze images by capturing fine details on a local level as well as the global context. In our work, we leverage the advantages of U-Net in capturing multi-scale features and preserving details through skip connections by incorporating gradient information and Kalman filter residues into a U-Net framework. U-Net’s intrinsic capacity to fuse various levels of abstraction makes it a suitable choice for merging multiple sources of information (noisy image, noise residue, and gradient) into one framework that guarantees both high-level denoising and the preservation of minute details.

**Fig. 1:**
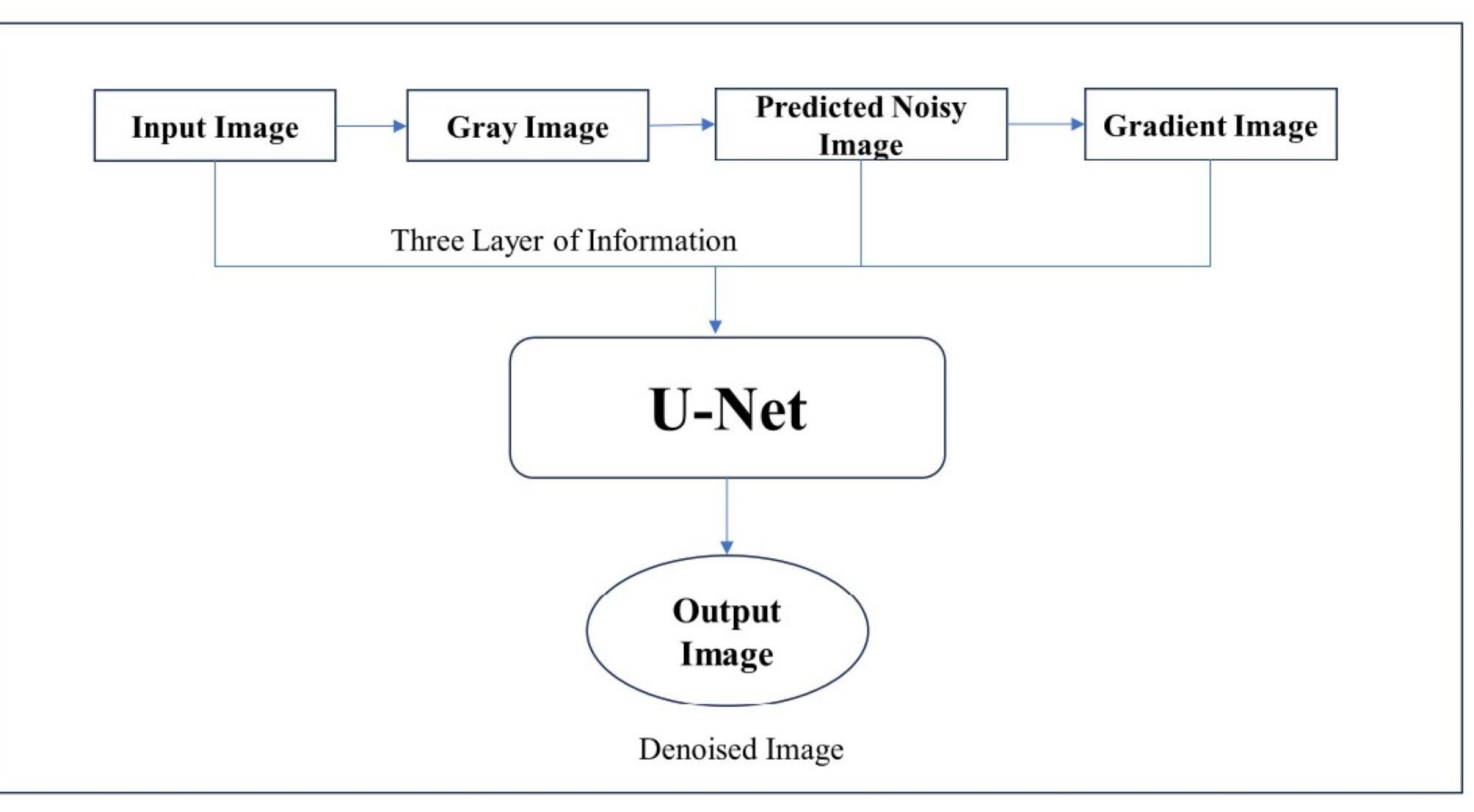
Design flow of KU-Net.

**Fig. 2:**
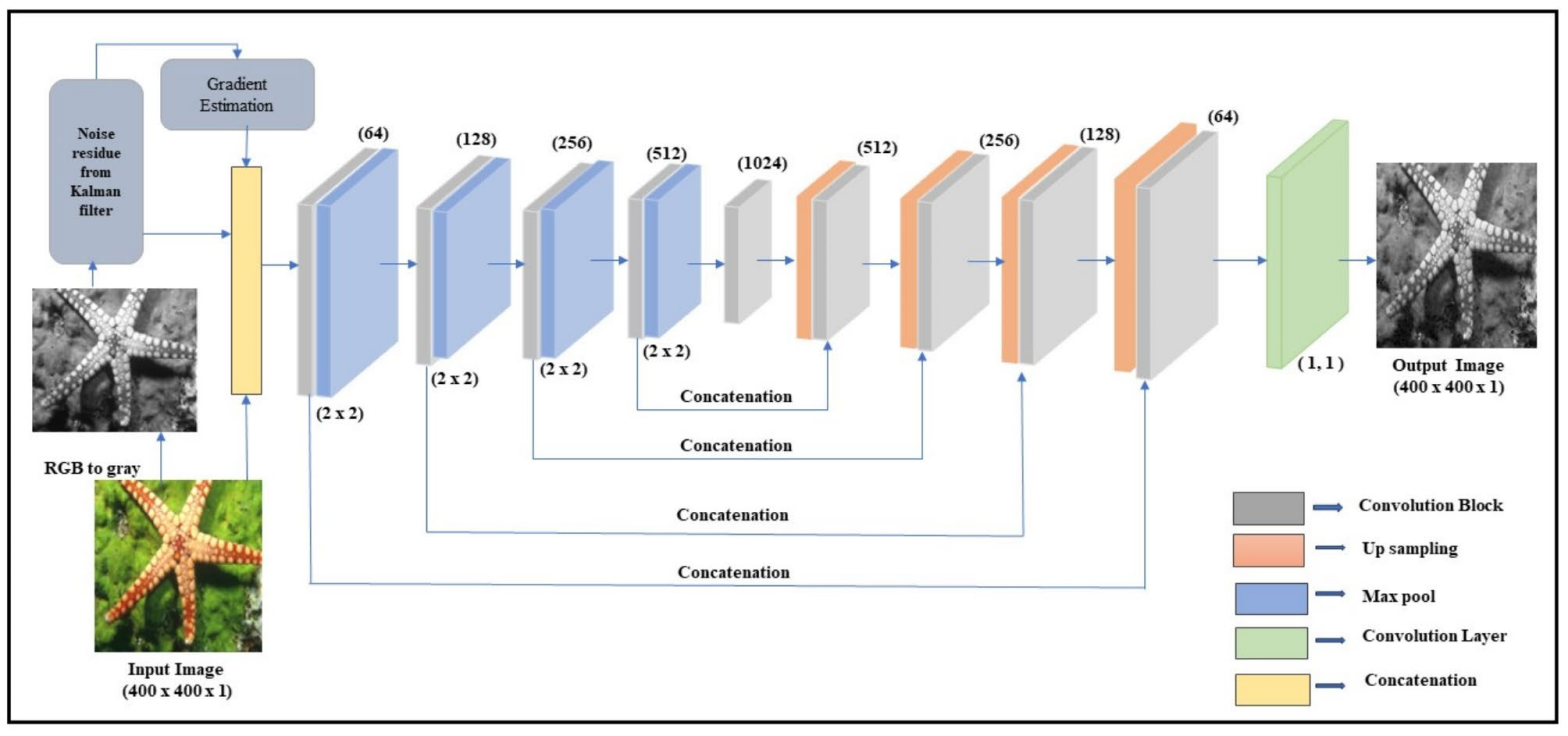
Design of proposed KU-Net architecture.

The high performance of the KU-Net architecture is achieved at the expense of simplicity compared to traditional models. Key components included in the architecture are as follows: The U-Net is made up of an encoder that uses downsampling to obtain context and a decoder that uses upsampling to reconstruct the image. Skip connections are used between corresponding layers of the encoder and decoder to maintain spatial information. This enables the network to fuse low-level detail features with high-level semantic information. The Kalman filter is utilized to estimate and eliminate noise from the input image. The U-Net receives the residue, which is the variation between the noisy image and the output of the Kalman filter. The residue furnishes details regarding the noise properties, allowing the network to modify its denoising approach. The incorporation of gradient estimation into the deep learning model was the primary innovation in this work. To extract texture and edge information from the noisy image, the gradient is computed. To maintain minute details while denoising, this gradient map is essential. Figure 1 represents the design flow of the KU-Net model, it describes the three layers of information applied to the U-Net then the encoder and decoder operate. Finally, a denoised image will produce at the output stage. Figure 2 shows how the suggested model is architecturally laid out.

The input RGB image is converted as a gray image with a size of 400 × 400. Initially, three layers of information are supplied to the U-Net architecture: input gray image, predicted noisy image, and gradient estimation. Consequently, two assessed image priors are concatenated with the input grayscale image. The U-Net structure’s encoding and decoding are planned to generate the denoised image based on this illustrated in Fig. 1. The encoders can perform down-sampling and convolution, which are accomplished by kernel filters and pooling layers, respectively. Decoders are constructed by concatenating encoder stage features and using up samplers, in contrast to encoders. Unlike the classification models, the final output is a denoised image rather than a class label.

The encoder and decoder consist of four convolutional blocks named B1, B2, B3, and B4. As illustrated in Fig. 2, the convolution blocks are used to construct the encoder and decoder sections. Two convolutional layers make up the internal structure of each convolution block. For downstream blocks 1, 2, 3, and 4, the number of filters in each encoder block is 64, 128, 256, and 512, respectively.

**Fig. 3:**
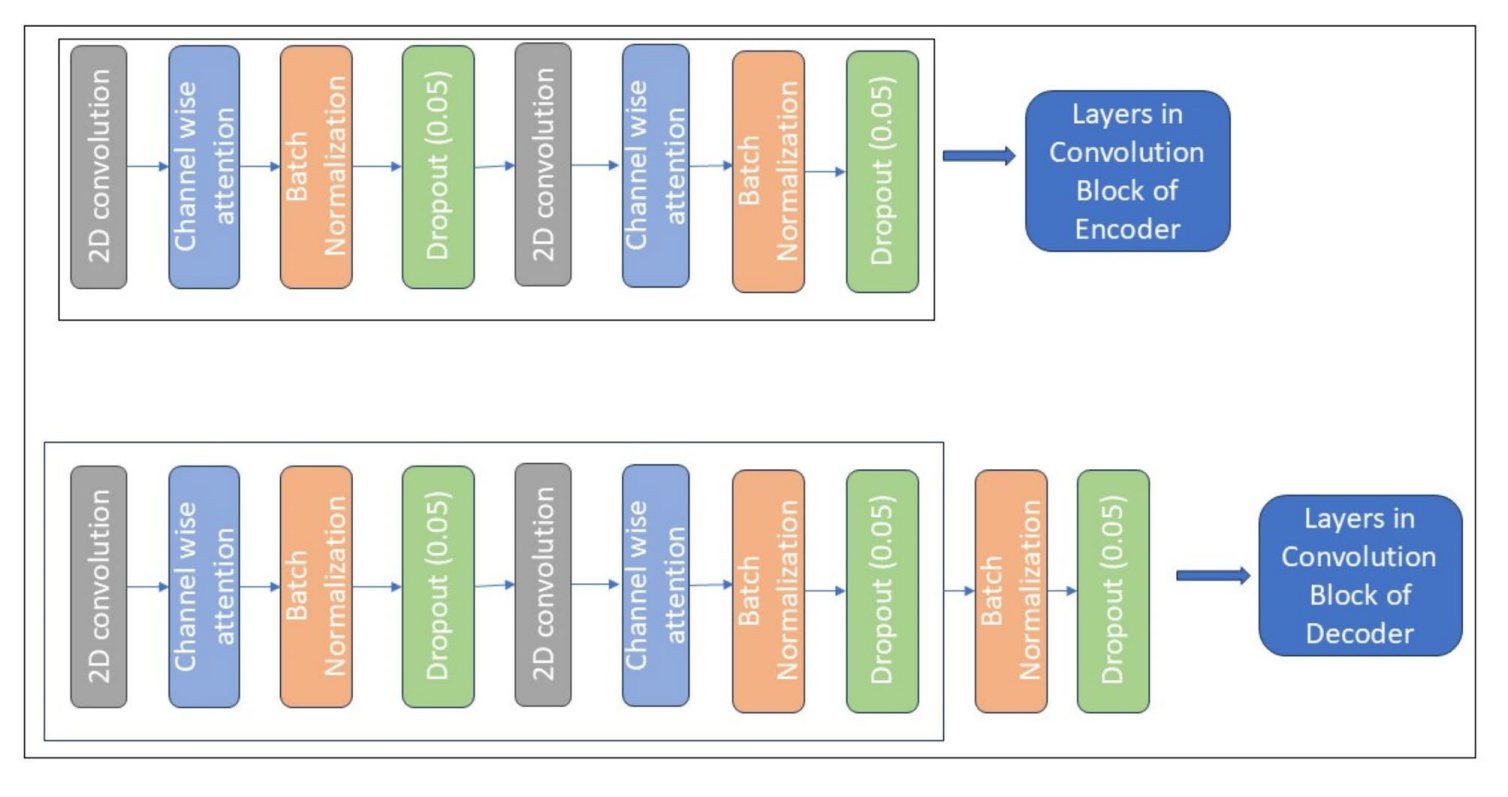
Convolution block layers in encoder and decoder.

Figure 3 depicts the encoder’s structure, which was used in this study. The activation function of the Rectified Linear Unit (ReLU) activation function activated every convolutional layer. Nevertheless, the final denoised image was produced by activating the final convolution layers using the “sigmoid” function. Drop-out layers were also incorporated into the convolutional layers of the encoder and decoder to enhance denoising performance. The stages of the encoder and decoder were dropped out at 0.05.

Let $\:Kn\:\left(m,n\right)$ be the denoised image and $\:K\left(m,n,\:\right)$ be the clean image represented in Eq. (1). Where (m, n) is the pixel location of the image.

1 $$\:Kn\:\left(m,n\right)=\:K\left(m,n\right)+\:Noise\left(0,\:\sigma\:\right)$$

Kalman filters predict the state of the system and estimate the previous state system. $\:{R}_{k}$provides the optimal noise-free pixel value and is thought to be a first-order AR model^34^. The behavior of pixels in an image is typically represented by this model. The process noise, denoted as $\:{n}_{k}$ and assumed to be white Gaussian with zero mean and variance $\:{\sigma\:}_{n}^{2}$, is represented by the constant and in the process model, which is dependent on the signal statistics.

2 $$\:{R}_{k+1}=a{R}_{k}+{n}_{k}$$

A 3 × 3 window is used to filter the Gaussian noise. Equation illustrates the approximate version of the noise matrix that is obtained by (3)

3 $$\:Noise\:\left(\:m,\:n\right)=\:{K}_{noisy}\left(m,\:n\right)-{K}_{denoised}\left(m,\:n\right)$$

Let $\:{K}_{d}\left(m,n\right)$ denote the extracted image obtained by applying the Kalman filter and the gradient magnitude image $\:{M}_{g}\left(m,n\right)$. Equation (6) mentions the gradient magnitude function.

4 $$\:{M}_{g}\left(m,n\right)=\:\sqrt{\left({{{M}_{g}}_{x}\left(\text{m},\text{n}\right)}^{2}\:+\:{{{M}_{g}}_{y}\left(\text{m},\text{n}\right)}^{2}\right)}\:$$

The vertical gradient $\:\:{{M}_{g}}_{y}\left(\text{m},\text{n}\right)\:\:$in (4b) and the horizontal gradient $\:{{M}_{g}}_{x}\left(\text{m},\text{n}\right)$ in (4a) are calculated using Sobel operators:

4a $$\begin{aligned} & {{M}_{g}}_{x}\left(\text{m},\text{n}\right)=\:\left({K}_{d}\left(m+1,\:n-1\right)+\:2\:{K}_{d}\:\left(m+1,\:n\right)+{K}_{d}\left(m+1,\:m+1\right)\right) \\ & \quad -\:\left({K}_{d}\:\left(m-1,\:n-1\right)+\:2{K}_{d}\:\left(m-1,\:n\right)+\:K\:\left(m-1,\:n+1\right)\right)\end{aligned}$$

4b $$\begin{aligned} & {{M}_{g}}_{y}\left(\text{m},\text{n}\right)=\:\left({K}_{d}\:\left(m-1,\:n-1\right)+\:2\:{K}_{d}\:\left(m,\:n-1\right)+\:{K}_{d}\:\left(m+1,\:n-1\right)\right) \\ & \quad -\:\left({K}_{d}\:\left(m-1,n+1\right)+\:2\:{K}_{d}\:\left(m,n+1\right)+{K}_{d}\:\left(m+1,\:n+1\right)\right) \end{aligned}$$

As a result, the model receives input from three layers of information: the input gray image, the noisy image predicted by Eq. (3), and the gradient information obtained by Eq. (4). After that, a denoised image is intended to be obtained through U-Net encoding and decoding.

### Metrics evaluation

The basic parameters are used to analyze the performance of denoising an image in the KU-Net architecture. The peak signal-to-noise ratio (PSNR) denotes a quality measurement of the original image and compressed image. The structural similarity Index (SSIM) provides a quantitative measure of edges and texture-aware information comparing two images. In this way, we consider the model performance in a denoising process.

The Mean Squared Error (MSE) loss function was selected because the problem involves reconstructing the noiseless image. During the training session, the loss function indicated in Eq. (5) was employed.

5 $$\:MSE\:loss=\:\frac{1}{M\:\times\:\:N}\:\sum\:_{i=1}^{M}\sum\:_{j=1}^{N}{\left({predicted}_{i,\:j}-{actual\:target}_{i,\:j}\:\right)}^{2}$$

In this case, $\:{actual\:target}_{i,\:j}$ = the original noise-free image; $\:{predicted}_{i,\:j}$= denoised images during training; and N = number of columns, M = number of rows. Reducing MSE loss and obtaining the least amount of error were the key performance indicators.

6 $$\:PSNR=\:10\text{log}\frac{{Max}^{2}}{MSE\:loss}\:\text{d}\text{B}$$

The testing performance was derived from the images that were not subjected to training and validation, where Max = Maximum value of the pixel in the original noise-free image. The average performance was determined to be 15, 25, and 50 dB in terms of MSE and PSNR, respectively.

7 $$\:SSIM\:\left(x,y\right)=\:\:\frac{\left(2{\mu\:}_{x}{\mu\:}_{y}+{c}_{1}\right)\left(2{\sigma\:}_{xy}+{c}_{2}\right)}{\left({\mu\:}_{x}^{2}+{\mu\:}_{y}^{2}+{c}_{1}\right)\left({\sigma\:}_{x}^{2}+{\sigma\:}_{y}^{2}+{c}_{2}\right)}$$

The similarity index between the two images indicates the SSIM values, which range [0,1]. Utilizing this metric, analyze the quality of the images.

8 $$\:FOM=\:\frac{1}{\text{m}\text{a}\text{x}\left(\left|{G}_{t}\right|\:\left|{D}_{c}\right|\right)}\sum\:\frac{1}{1+k.{d}_{{G}_{t}}^{2}\left(p\right)}$$

The quality of edge detection in images is assessed using a metric called the Figure of Merit (FOM). It evaluates the position and magnitude of the detected edges as well as the accuracy of the detected edges about a ground truth.

### Parameter settings

Initially, 1 × 10^− 3^ was the model’s learning rate. Adaptively, this rate was changed in response to the validation performance. The learning rate was lowered in non-linear steps of 1 × 10^− 5^, 1 × 10^− 6^, and the least of 1 × 10^− 7^. The value for L2 regularisation was 0.000001. When ten patient attempts were made and there was no improvement, this adaptive variation was carried out using the call-back functions. The Adam optimizer was used to optimize the model^35^. For the BSD300 database, the batch size was changed to 4. Fifty epochs were used to train this model. To cover all training images, the sessions in the training loop were iterated through 4 images per batch. In this work, image augmentation was not done because the primary goal is to enhance the traditional U-Net model’s learning capability.

### Datasets

**Fig. 4:**
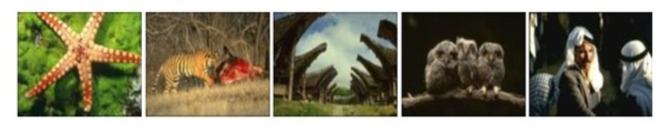
Sample images from the BSD dataset.

This paper adopted different types of datasets that are available in online sources, and for training purposes, the Berkeley Segmentation Dataset (BSD) has been chosen. There are a total of 300 images split as 240 images for training and 30/30 images for validating and testing with the dimensions of 481 × 321 and 321 × 481 with three colors of R, G, and B. The images were resized as 400 × 400, with an option of nearest interpolation. After randomly arranging the image names into 42 states, the database was divided. The original scale for each image was changed from [0, 255] to within the range of [0, 1]. Also, for comprehensive experimental results comparison Set12, CBSD68, KODAK24, and McMaster datasets are proceeded. For the BSD dataset, sample images are displayed in Fig. 4. Further, we tested our model by using four different datasets they are Set12, KODAK24, McMaster, and CBSD68.

### Experimental setup

Our model was trained on an NVIDIA GeForce GTX1650 graphical processing unit (GPU), and Collab was used to conduct all of the experiments on a PC equipped with an Intel Core i5-10300 H processor and 8 GB of RAM.

### Time complexity analysis

The time complexity analysis of our proposed work KU-Net is calculated by the approximation of summing the number of Floating-point operations (FLOPs) required for the Deconvolution and Convolution layer. The total computational complexity for the given KU-Net model, with an input size of 256 × 256 × 1, a kernel size of 3 × 3, and a variable number of filters through the network, is computed as follows: For Encoder block-891,550,720 operations, Bottleneck layer-3,220,758,528 operations, Decoder block-1,476,917,376 operations, and Output Layer-4,194,304 operations. Finally, summing these the total time complexity of the model is 5,593,420,928 approximately 5.6 × 10^9^ operations. The FLOPs per second for our NVIDIA V100 GPU is rounded as 5.6 GFLOPS/s (gigaflops per second).

## Results and discussion

In this article, we designed a novel network called KU-Net and particularly, aimed to construct a simple architecture and achieve a better denoising performance compared to the complex architectures. In Table 2, PSNR, SSIM, and FOM indices are used to evaluate the performance of the model at the noise level of 15, 25 and 50.

**Table 2:** Basic metrics evaluation.

Metrics	σ = 15	σ = 25	σ = 50
PSNR (dB)	31.04	29.38	26.60
SSIM	0.9432	0.8985	0.7995
FOM	0.9123	0.8828	0.8485

We assess KU-Net’s performance by contrasting it with state-of-the-art techniques for image denoising, such as model-based and learning-based approaches. We use noise sigmas of 15, 25, and 50 standard deviations to corrupt clean photos, and then we use these noisy grayscale images to test our model. We contrast our approach with various denoising algorithms. Table 3 reveals the denoising performance of the proposed work with a comparison of various traditional methods like BM3D^11^, WNNM^36^, EPLL^37^, CSF^38^, MLP^1^, TNRD^12^, IRCNN^15^, DnCNN^14^, FFDNet^13^ and BRDNet^19^. We use the average structural similarity index (SSIM) and the peak signal-to-noise ratio (PSNR) to evaluate the prior models. To facilitate comparison, we present the findings for the most often utilized noise levels in the literature, σ = {15, 25, and 50}.

**Table 3:** Average PSNR value of traditional methods compared to the proposed model.

Methods	BM3D ^11^	WNNM ^24^	EPLL ^25^	CSF ^26^	MLP ^1^	TNRD ^12^	IRCNN ^15^	DnCNN ^14^	FFDNet ^13^	BRDNet ^19^	KU-Net
σ = 50	25.62	25.87	25.67	–	26.03	25.97	26.19	26.23	26.30	26.36	26.60
σ = 25	28.57	28.83	28.68	28.74	28.96	28.92	29.15	29.23	29.19	29.29	29.38
σ = 15	31.07	31.37	31.21	31.24	–	31.42	31.63	31.72	31.62	31.79	31.04

**Fig. 5:**
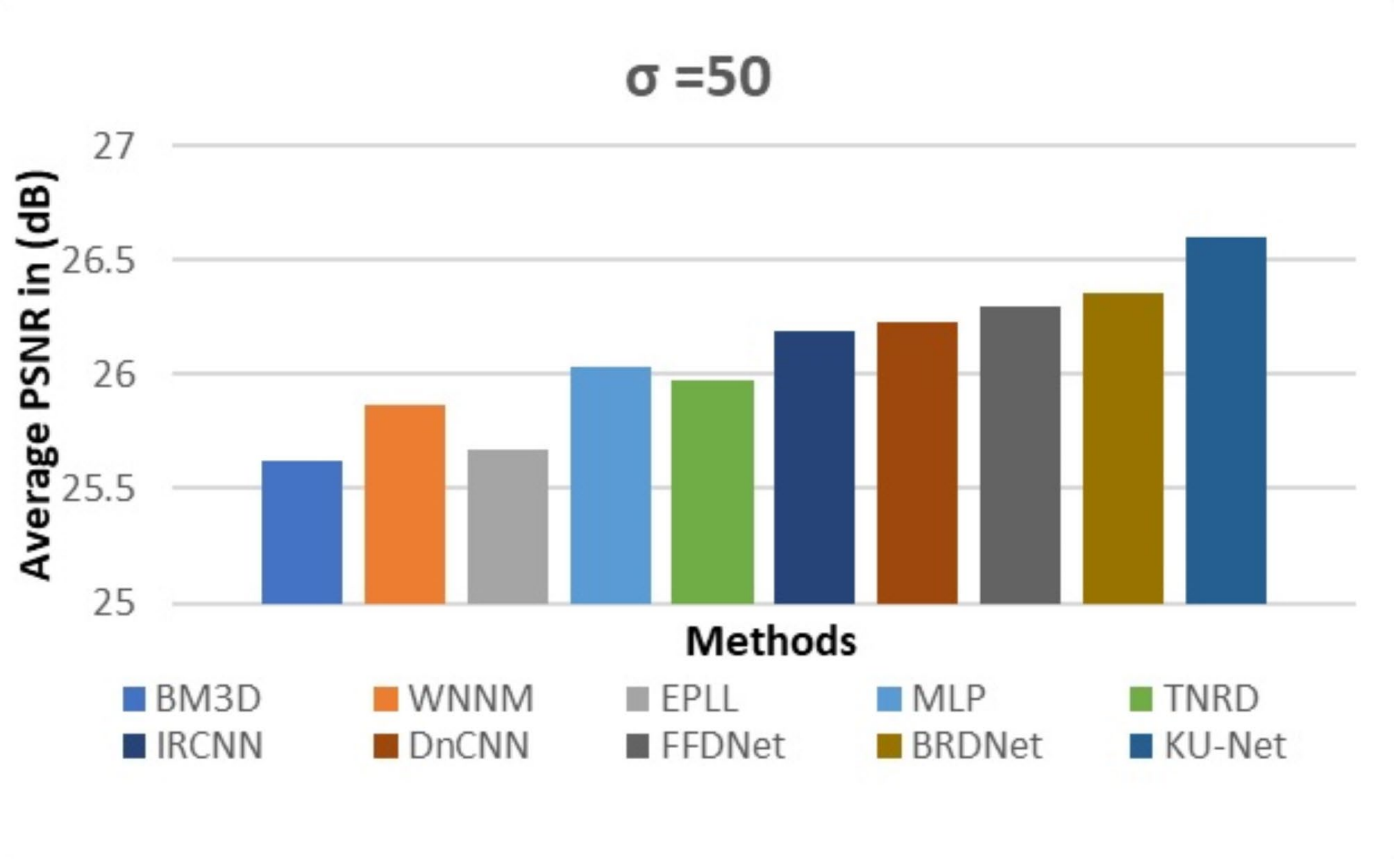
Performance analysis of proposed KU-Net with other models.

By evaluating the proposed method, Fig. 5 represents the comparative analysis that has referenced different traditional methods. It is observed that KU-Net architecture provides a better result.

**Fig. 6:**
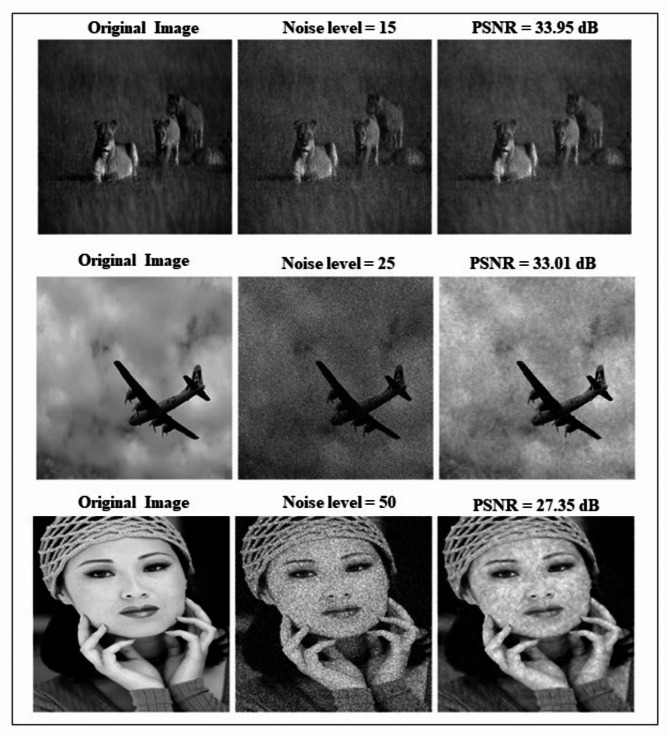
Denoising performance of KU-Net for selected image in BSD database.

The Peak Signal to Noise Ratio of a single image is described in Fig. 6 when compared to the original image, the output image had more similarity. The below images are taken from the BSD dataset and analyzed a three different noise levels of 50, 25, and 15 respectively. For σ = 15, PSNR = 33.95 dB, for σ = 25, PSNR = 33.01dB and for σ = 50, PSNR = 27.35 dB. We attempt a different image for different noise levels in contrast with the original image. When we examine the visualized results, we discover that the denoised images generated by our suggested model are the most similar to the original images in terms of information and sharpness of edges.

**Fig. 7:**
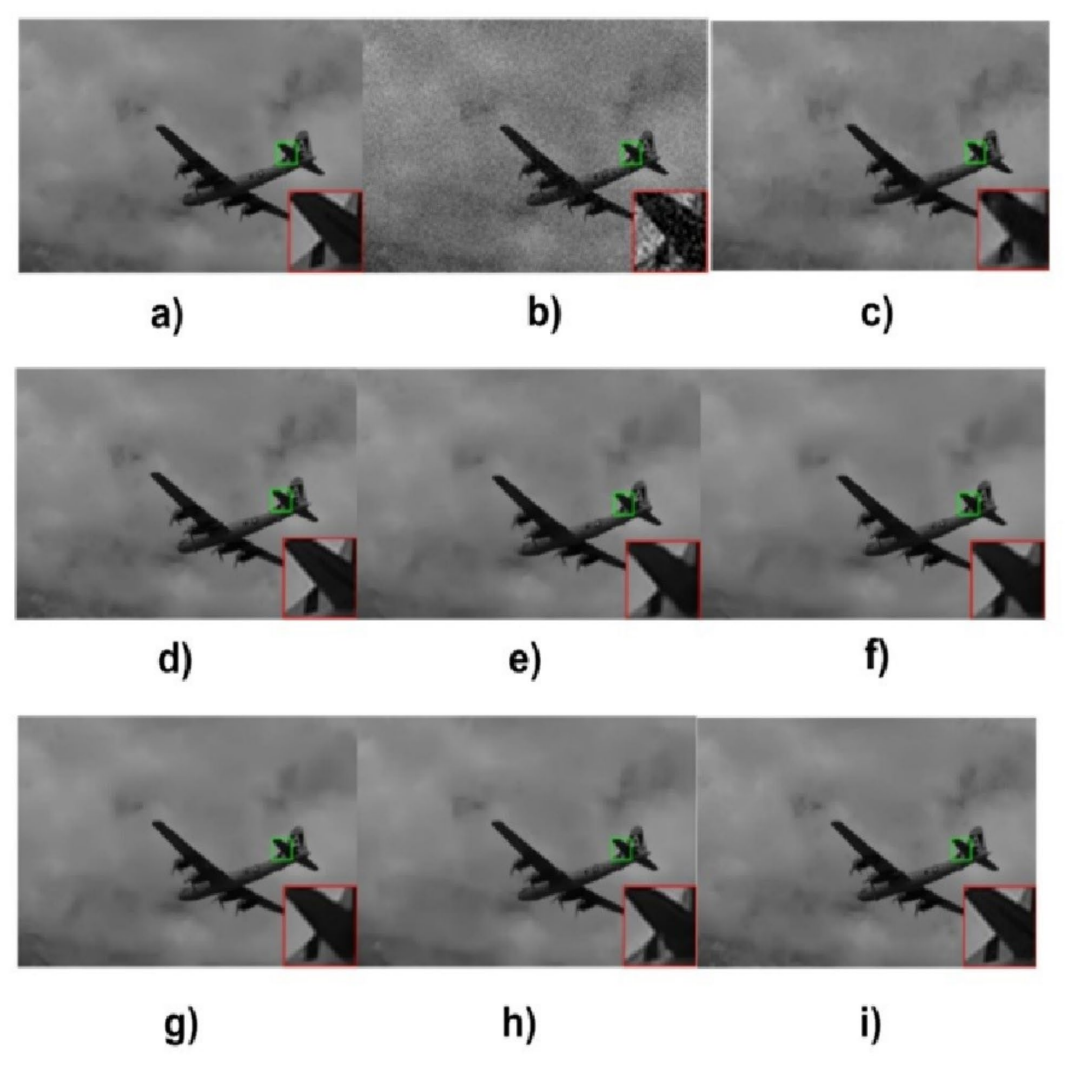
Visual comparison of KU-Net with state-of-the-art methods.

A set of graphic comparisons to show off the proposed KU-Net method’s efficacy. We compare the denoised images obtained from our model with the outcomes of other cutting-edge denoising techniques. In Fig. 7, flight images from the BSD dataset are used. By evaluating the performance, the image PSNR value of (i) KU-Net is higher at 38.85 dB compared with the (c) BM3D^11^/36.59 dB, (d) WNNM^36^/37.22 dB, (e) IRCNN^15^/38.17 dB, (f) FFDNet^13^/38.41 dB, (g) DnCNN^14^/38.45 dB, (h) RDDCNN^39^/38.64 dB and (a) Original image & (b) Noisy image/20.19 dB.

Denoising an image primarily concentrates on image content processing. We tested our work with the Set12 dataset for the noise level of 50, 25, and 15. Table 4 shows the average PSNR value of every model and describes each image value. From the comparison table, we conclude that our KU-Net architecture provides better performance in noise levels of 50 and 25, with slight differences in 15. We plan to work on lower noise levels and color image denoising in future work.

**Table 4:** Average PSNR value of each image in the Set12 dataset on different methods with the noise level of 15, 25, and 50.

Images	BM3D	TNRD	FFDNet	DnCNN	IRCNN	DudeNet	McCNN	ADNet	BRDNet	MLFAN	KU-Net
	^11^	^12^	^13^	^14^	^15^	^16^	^17^	^18^	^19^	^20^	
σ = 50 (Noise level)											
Barbara	27.22	25.70	26.45	26.22	26.24	26.49	26.53	26.64	26.85	26.96	26.98
Starfish	25.04	25.42	25.75	25.70	25.57	25.88	25.89	25.70	25.77	25.97	25.87
C.man	26.13	26.62	27.05	27.03	26.88	27.22	27.13	27.31	27.44	27.53	27.61
Lena	29.05	28.93	29.66	29.39	29.40	29.45	29.62	29.59	29.73	29.75	29.76
Boat	26.78	26.94	27.33	27.20	27.17	27.26	27.29	27.35	27.38	27.44	27.49
Man	26.81	26.98	27.29	27.24	27.17	27.19	27.31	27.17	27.27	27.36	27.43
House	29.69	29.48	30.37	30.00	29.96	30.27	30.33	30.59	30.53	30.90	30.98
Couple	26.46	26.50	27.08	26.90	26.88	26.97	27.13	27.07	27.17	27.36	27.39
Peppers	26.68	27.10	27.54	27.32	27.33	27.51	27.46	27.69	27.67	27.69	27.74
Airplane	25.10	25.59	25.89	25.87	25.89	25.88	25.94	25.88	25.93	26.09	26.26
Monarch	25.82	26.31	26.81	26.78	26.61	26.93	26.91	26.90	26.97	27.06	27.22
Parrot	25.90	26.16	26.57	26.48	26.55	26.50	26.59	26.56	26.66	26.64	26.71
Average	26.72	26.81	27.32	27.18	27.14	27.30	27.34	27.37	27.45	27.56	27.62
σ = 25 (Noise level)											
Barbara	30.71	29.41	30.01	30.00	29.92	30.15	30.20	30.25	30.34	30.41	30.44
Starfish	28.56	29.02	29.32	29.41	29.27	29.53	29.48	29.41	29.46	29.67	29.86
C.man	29.45	29.72	30.10	30.18	30.08	30.23	30.30	30.34	31.39	30.53	30.03
Lena	32.07	32.00	32.57	32.44	32.43	32.52	32.55	32.61	32.65	32.75	32.79
Boat	29.90	29.91	30.25	30.21	30.17	30.24	30.27	30.37	30.33	30.42	30.52
Man	29.61	29.87	30.11	30.10	30.04	30.08	30.14	30.08	30.14	30.18	30.20
House	32.85	32.53	33.28	33.06	33.06	33.24	33.39	33.41	33.41	33.79	33.81
Couple	29.71	29.71	30.20	30.12	30.08	30.15	30.19	30.24	30.28	30.41	30.33
Peppers	30.16	30.57	30.93	30.87	30.88	30.98	30.93	31.14	31.04	31.18	31.21
Airplane	28.42	28.88	29.04	29.13	29.12	29.14	29.18	29.17	29.20	29.34	29.41
Monarch	29.25	29.85	30.08	30.28	30.09	30.44	30.47	30.39	30.50	30.61	30.79
Parrot	28.93	29.18	29.44	29.43	29.47	29.48	29.43	29.49	29.55	29.66	29.75
Average	29.97	30.06	30.44	30.43	30.38	30.52	30.54	30.58	30.61	30.75	30.76
σ = 15 (Noise level)											
Barbara	33.10	32.13	32.54	32.64	32.43	32.73	32.69	32.80	32.93	32.86	31.92
Starfish	31.14	31.75	31.99	32.20	32.02	32.29	32.29	32.17	32.24	32.36	32.01
C.man	31.91	32.19	32.43	32.61	32.55	32.71	32.65	32.81	32.80	32.88	31.89
Lena	34.26	34.24	34.62	34.62	34.53	34.66	34.65	34.71	34.75	34.78	33.98
Boat	32.13	32.14	32.38	32.42	32.34	32.46	32.47	32.57	32.55	32.61	31.85
Man	31.92	32.23	32.41	32.46	32.40	32.46	32.44	32.47	32.50	32.53	31.97
House	34.93	34.53	35.07	34.97	34.89	35.13	35.20	35.22	35.27	35.39	33.67
Couple	32.10	32.11	32.46	32.47	32.40	32.49	32.53	35.22	32.62	32.68	32.12
Peppers	32.69	33.04	33.25	33.30	33.31	33.38	33.35	33.49	33.47	33.59	32.78
Airplane	31.07	31.46	31.57	31.70	31.70	31.78	31.78	31.86	31.85	31.92	30.56
Monarch	31.85	32.56	32.66	33.09	32.82	33.28	33.30	33.17	33.35	33.33	32.42
Parrot	31.37	31.63	31.81	31.83	31.84	31.93	31.98	31.96	32.00	32.05	31.33
Average	32.37	32.50	32.77	32.86	32.77	32.94	32.94	32.98	33.03	33.08	32.20

**Fig. 8:**
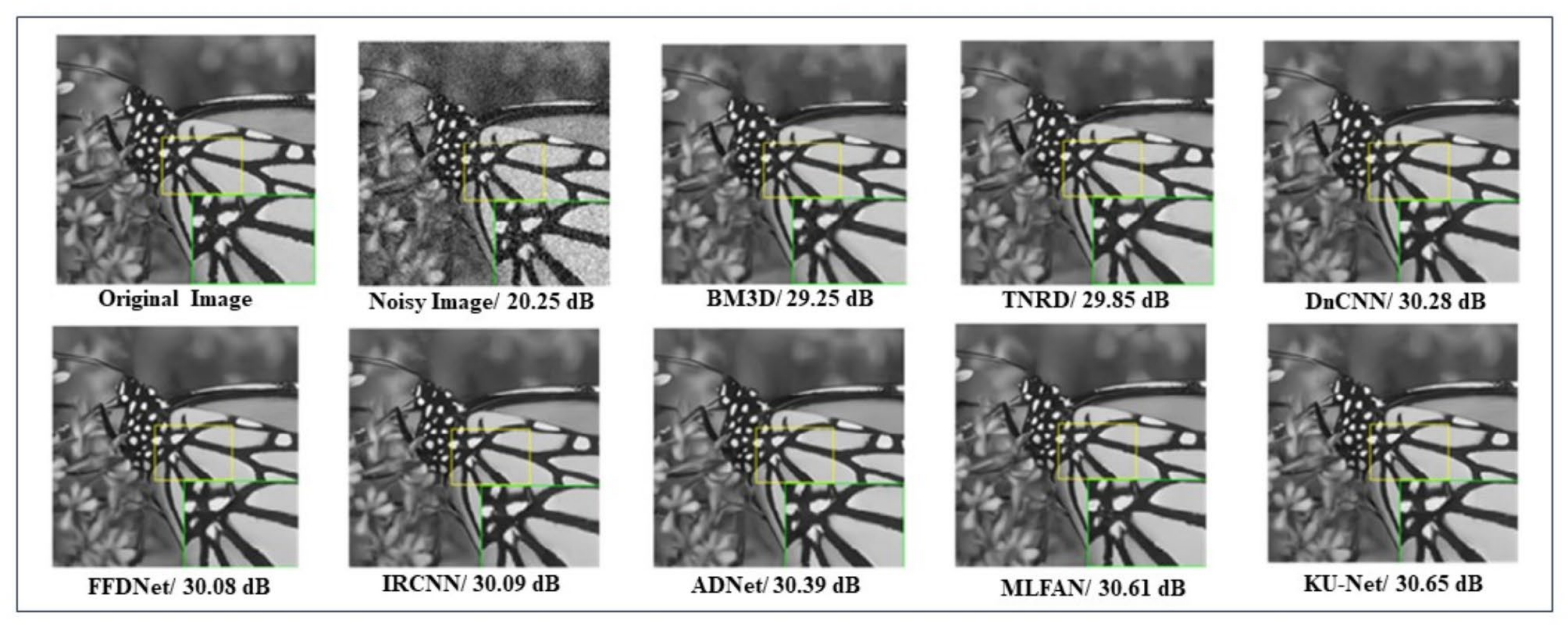
Denoising results of Single image (Monarch) in Set12 dataset with the noise level of 25.

In Fig. 8, we analyze a single image of Monarch from the Set12 dataset with a noise level of 25. The process of denoising an image in KU-Net architecture yields 30.65 dB of PSNR value, comparison model images also are listed below.

By analyzing performance, a lot of architectures are evolving and moving towards a recent trend. In this paper, we concentrate on the achievement of previous models and obtain more datasets that are available online and links are also provided in data availability statements. Table 5 demonstrates the values of PSNR and SSIM values. For testing purposes, we take CBSD68, Kodak24, and McMaster and evaluate with six complex architectures. According to experiments on multiple datasets, our suggested network outperforms traditional methods and is competitive with state-of-the-art techniques.

**Table 5:** Comparative analysis of different datasets on various models.

Datasets	Models	PSNR in dB			SSIM		
		σ = 15	σ = 25	σ = 50	σ = 15	σ = 25	σ = 50
CBSD68	CBM3D ^11^	33.52	30.71	27.38	0.9248	0.8716	0.7669
	DnCNN ^14^	33.98	31.31	28.01	0.9317	0.8863	0.7915
	FFDNet ^13^	33.80	31.18	27.96	0.9318	0.8857	0.7916
	IRCNN ^15^	33.86	31.16	27.86	0.9285	0.8824	0.7898
	ADNet ^18^	33.99	31.31	28.04	0.9334	0.8889	0.7977
	MLFAN^20^	34.06	31.37	28.11	0.9336	0.8894	0.7982
	KU- Net	32.45	31.38	28.22	0.9332	0.8896	0.7989
Kodak24	CBM3D^11^	34.28	31.68	28.46	0.9164	0.8682	0.7751
	DnCNN^14^	34.73	32.23	29.02	0.9212	0.8774	0.7895
	FFDNet^13^	34.55	32.11	28.99	0.9230	0.8790	0.7929
	IRCNN^15^	34.56	32.03	28.81	0.9198	0.8766	0.7929
	ADNet^18^	34.76	32.26	29.10	0.9248	0.8830	0.7997
	MLFAN^20^	34.85	32.33	29.15	0.9257	0.8835	0.8000
	KU- Net	33.65	32.36	29.17	0.9249	0.8835	0.8001
McMaster	CBM3D^11^	34.06	31.66	28.51	0.9150	0.8739	0.7934
	DnCNN^14^	34.8	32.47	29.21	0.9070	0.8724	0.7985
	FFDNet^13^	34.47	32.25	29.14	0.9247	0.8891	0.8157
	IRCNN^15^	34.58	32.18	28.91	0.9195	0.8818	0.8069
	ADNet^18^	34.93	32.56	29.36	0.9287	0.8944	0.8246
	MLFAN^20^	35.08	32.68	29.47	0.9288	0.8956	0.8263
	KU- Net	33.97	32.69	29.50	0.9279	0.8958	0.8266

To boost the model’s performance even more, we have evolved several significant methods. Our model thus attains the best visualization results in image denoising. This demonstrates the practicality of applying our model. Additionally, these methods ought to serve as inspiration for similar assignments.

### Running time

**Fig. 9:**
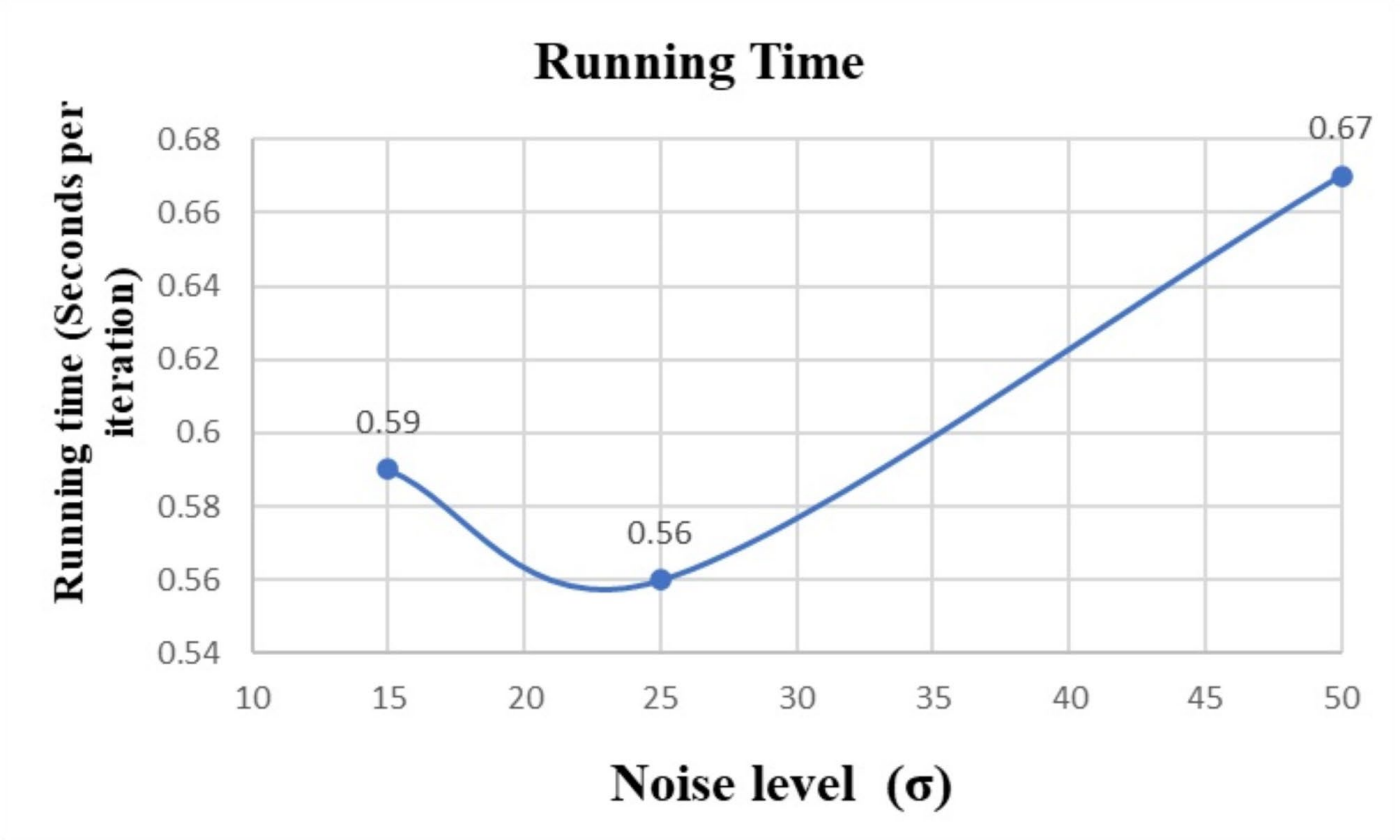
Running time with noise densities.

Additional experiments have been carried out to analyze the running time under various noise densities to further demonstrate the performance of KU-Net. We have added a curve that shows the relationship between running time and noise density to help visualize the performance of the suggested method in different noise scenarios in Fig. 9. The efficiency and scalability of the approach across a range of noise levels can be better understood with the help of this curve.

By calculating the running time of our proposed model using the formula, Running time (sec per iteration) = 1/iterations per second. For each noise level of 15, 25, and 50; the speed is 1.67,1.78, and 1.49 iterations per second. Therefore, the running time of noise level 15 is 0.59 s, 25 is 0.56 s, and 50 is 0.67 s.

### Real-time denoising

The model is well suited for deployment in mobile and embedded systems where computational resources are limited because of its simplicity and effective denoising capabilities. Camera software could produce clear, high-quality images even in low light by utilizing KU-Net to process noisy images in real-time or almost real-time without appreciably increasing computational overhead.

KU-Net is better suited for deployment on edge devices, such as smartphones, which have limited power and storage, due to its more straightforward structure and emphasis on computational efficiency. Our proposed work is suitable for real-time applications because it has fewer parameters than more complicated architectures, which would enable it to operate more quickly while consuming less memory and processing power. In low light, noise reduction on smartphones is usually a problem. The performance of “night mode” and other low-light photography features on smartphones and cameras can be improved with KU-Net’s ability to denoise while maintaining details, resulting in sharper and more detailed images.

Manually modifying photo editing or noise reduction settings is not always intuitive for the typical consumer. Users might receive better, cleaner images from KU-Net automatically, saving them from labour-intensive post-capture editing.

When recording fast-moving objects or in low light, noise can be particularly distracting in videography. Videos could become smoother and cleaner if KU-Net reduced video noise on a frame-by-frame basis. This ensures better quality without requiring the purchase of expensive equipment, which is especially helpful for amateur videographers who use their cell phones to capture everyday moments.

KU-Net can assist in preserving temporal consistency during the denoising process, which could lessen the flickering that occasionally happens with conventional denoising techniques. This helps videos appear steadier and more polished, even when they are taken in difficult lighting circumstances.

## Conclusion

The use of Kalman filtering in the U-Net architecture is a significant innovation of this work. The network makes use of the estimated noise residues from the Kalman filter to achieve more accurate denoising. This method is especially new for deep learning-based denoising, where these kinds of signal processing methods are rarely used but provide substantial advantages in noise estimation precision. To enhance the preservation of edges and textures during the denoising process, gradient information is explicitly incorporated into the KU-Net model. Fine details in the image are preserved by KU-Net by feeding the gradient estimation as one of the network’s inputs, which are frequently lost in conventional denoising techniques. The ability to preserve the denoised image’s visual quality is especially helpful.

KU-Net has shown exceptional performance in image-denoising tasks through extensive experiments, especially at higher noise levels. Even with limited datasets for training, the model outperforms several State-of-the-Art methods in terms of PSNR and SSIM values. At a higher noise level, the proposed model outperforms by gaining a PSNR value of 26.60 dB with a noise level of σ = 50. Highly complex models are not always necessary to achieve high-quality denoising; as demonstrated by KU-Net, which has a simpler architecture but still achieves competitive or even superior performance. Although KU-Net has many benefits, the paper also critically looks at its drawbacks. At the lower noise level of 15, the comparative analysis yielded values that were closer to those of the current models. We intend to work with color, realistically noisy images, and improved performance in lower-noise environments in the future. We also intend to discuss possible directions for future research, including investigating larger datasets, using the model for different image processing applications, and refining the noise residue and gradient information integration.

## Data Availability

For Training: BSD dataset-https://www2.eecs.berkeley.edu/Research/Projects/CS/vision/bsds/. For Testing: SET12, CBSD68, KODAK24, and McMaster datasets used. The testing dataset was obtained from Kaggle and uploaded to Figshare. A link to the dataset is provided for easy access. 10.6084/m9.figshare.26827765.v1.
